# Viewing systematic reviews and meta-analysis in social research through different lenses

**DOI:** 10.1186/2193-1801-3-511

**Published:** 2014-09-10

**Authors:** Jacqueline Davis, Kerrie Mengersen, Sarah Bennett, Lorraine Mazerolle

**Affiliations:** Institute for Social Science Research, University of Queensland, Brisbane, St Lucia 4072 Australia; School of Mathematical Sciences, Queensland University of Technology, GPO Box 2434, Brisbane, 4001 Australia

**Keywords:** Meta-analysis, Heterogeneity, Systematic review, Missing data

## Abstract

Systematic reviews and meta-analyses are used to combine results across studies to determine an overall effect. Meta-analysis is especially useful for combining evidence to inform social policy, but meta-analyses of applied social science research may encounter practical issues arising from the nature of the research domain. The current paper identifies potential resolutions to four issues that may be encountered in systematic reviews and meta-analyses in social research. The four issues are: scoping and targeting research questions appropriate for meta-analysis; selecting eligibility criteria where primary studies vary in research design and choice of outcome measures; dealing with inconsistent reporting in primary studies; and identifying sources of heterogeneity with multiple confounded moderators. The paper presents an overview of each issue with a review of potential resolutions, identified from similar issues encountered in meta-analysis in medical and biological sciences. The discussion aims to share and improve methodology in systematic reviews and meta-analysis by promoting cross-disciplinary communication, that is, to encourage ‘viewing through different lenses’.

## Background

Systematic reviews and meta-analyses are increasingly important techniques in social science research. These techniques are used to synthesise research results to determine an overall effect estimate for a population of studies. A systematic review refers to the process of systematically locating and collating all available information on an effect. Meta-analysis refers to the statistical techniques used to combine this information to give an overall estimate of the effect in the population. Together, systematic reviews and meta-analyses can help to clarify the state of a field of research, determine whether an effect is constant across studies, and discover what future studies are required to demonstrate the effect. Advanced meta-analysis techniques can also be used to discover what study-level or sample characteristics have an effect on the phenomenon being studied; for example, whether studies conducted in one cultural context show significantly different results from studies conducted in other cultural contexts.

**Figure 1:**
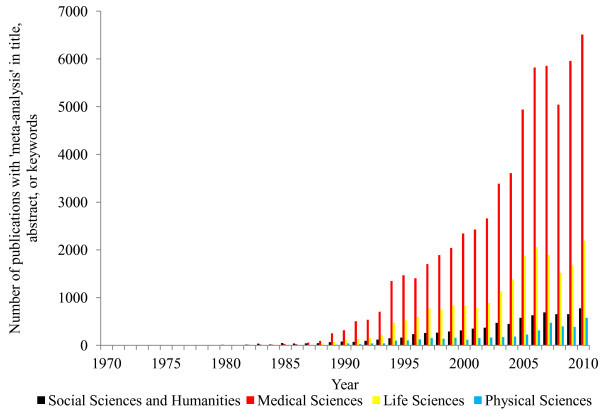
**Results of a Scopus search for “meta-analysis in title, abstract and keywords.**

Although meta-analysis was originally devised for use in the social sciences (Glass, 1976), the technique was quickly adopted and further developed for use in the medical sciences. Currently the medical sciences produce the majority of the literature on meta-analysis, including meta-analysis methods. In the social sciences, the use of meta-analysis is rapidly increasing (Figure 1), with meta-analysis being applied to an ever-broader range of subject matter. The application of meta-analysis to social science research, however, is not necessarily straightforward, and methods developed in medical research may be difficult to access and apply to social research, especially for applied researchers seeking to use meta-analysis in their own disciplines for the first time.

A number of techniques and processes, each requiring methodological choices, fall under the umbrella term ‘meta-analysis’. With the diversity of new applications for meta-analysis, new issues in implementing the methodology have arisen. Some of these issues have been addressed by review co-coordinating bodies, and recommendations have been made on how to deal with them; for example, the issue of publication or small-study bias has been carefully addressed (Higgins & Green, 2011). Other problems seem to have been raised independently in different disciplines, with a lack of overarching consensus on how to resolve them, and individual study authors applying ad hoc resolutions as they encounter each issue. Indeed, it is difficult for even experienced meta-analysts to follow ongoing methodological and technical debates and keep up with latest findings, especially across different substantive disciplines (Schulze, 2007). This lack of communication is particularly acute in disciplines that have only recently begun to use meta-analysis and where research data are less structured than in clinical disciplines. In these cases, researchers may look across disciplines, to view meta-analysis through other disciplinary lenses, and see the similarity between issues encountered in their own reviews and issues that have been encountered, and addressed, in the work of others.

The current paper reviews four practical issues that may be encountered in meta-analyses of applied social science research, and presents a multidisciplinary review of some approaches that have been used to resolve these. The first issue is scoping and targeting the systematic review to ensure that the question is appropriate for meta-analysis. The second is choosing eligibility criteria for included studies, in the absence of consensus on valid evaluation designs and appropriate outcome measures within the primary studies. The third is dealing with inconsistent reporting styles in the body of primary research, which greatly increase the difficulty of meta-analysis, any analysis of heterogeneity, and the application of any statistical tests or corrections. The final issue is attempting moderator analysis in the presence of multiple confounded study-level moderators.

The intent of the following sections is to provide context and guidance to applied researchers seeking to use meta-analysis to synthesise research in their own domains, to inform their own research or to provide guidance for social policy. Each issue is presented with a brief description and an example, followed by options for addressing the issue, with an effort to include alternatives from multiple academic disciplines. This discussion is not intended to provide a full guide to meta-analysis, but instead, to highlight areas of research that may offer assistance to reviewers encountering one or more of these issues in the process of their own systematic review and meta-analysis.

## Results and discussion

### Issue 1. Scoping and targeting the review

Meta-analysis having been defined as a “gold standard” for evidence-based practice in medicine (Sackett et al. 2000), and the increasing number of meta-analyses on a wide variety of topics, may give the impression that meta-analysis is the best technique to answer any research question in any field. Meta-analysis is, however, a specific statistical technique, and like any statistical technique is only appropriate for a narrow range of research questions and data. Scoping decisions have been addressed elsewhere, including choosing between broad and narrow inclusion criteria (see Issue 2, below), and whether to take a “black box” effectiveness-led approach or to focus on the specific causal pathways of an intervention (Stewart et al. 2012). A further, less well-addressed, issue is scoping a meta-analysis in a research area dominated by few large-scale empirical studies.

Many fields, including ecology, medicine, public health, and criminology, face the problem of a single large study dominating a particular field of research. Manipulating policy may be a practically and ethically difficult, and so tests of policy effectiveness may come in the form of quasi-experimental studies where the comparison group is a different geographical area, different time point, or waiting list. When a randomised field trial is conducted, it may be of very large scale compared to non-randomised evaluations of the same intervention, because of the resources required to establish agency partnerships; for example, initiating a field trial of a policing strategy requires cooperation of many agencies, including police officers, police management, multiple levels of government, as well as administrative and funding bodies. The dominance of such large-scale trials can result in a broad area of literature being based on very few independent studies, with multiple scientific articles resulting from the same dataset.

A possible result of this dominant study phenomenon is a disproportionate sense of optimism around the strength of evidence for a particular intervention or theory. Many papers stemming from few empirical studies is problematic for meta-analysis because the technique requires each observation included in the meta-analysis to be independent, so the true number of effect sizes available for meta-analysis may be much smaller than it first appears. Further problems can arise when the large-scale trials are systematically different to smaller trials, due to the different requirements of each; for example, when randomised trials are large and non-randomised trials are small, it may be difficult to tell whether differences in results are due to the size of the trial or the randomisation.

An illustration of the dominant study issue is given by Mazerolle et al. ( 2013) in a legitimacy policing review. A number of observational surveys, combined with multiple publications reporting on a large-scale field trial (Sherman et al. 1998) and a great deal of theoretical debate, produced an impression of a large amount of evidence supporting the effectiveness of legitimacy interventions in policing. When an attempt was made to locate this evidence, however, few studies were identified that tested actual interventions, and very few were randomised. The authors resolved their issue by developing a tailored set of standards for eligible comparison groups, including study design in a moderator analysis, and proceeding with the meta-analysis. However, other reviewers may have decided that the available evidence was not sufficient for meta-analysis.

Deciding whether evidence is suitable for meta-analysis is, at present, a question of judgement, and what is suitable evidence in one discipline may be unacceptable in another (Koricheva et al. 2013: Ioannidis et al. 2008). Questions for which meta-analysis is not suited may be better addressed using a traditional narrative review or an alternative, such as best-evidence synthesis (Slavin 1987), thematic synthesis (Thomas & Harden, 2008), interpretive synthesis (Dixon-Woods et al. 2006), or scoping reviews (Arksey & O’Malley, 2005). These techniques may also be used as a broader background to a more focused meta-analysis, enabling both a broad review of a field and a statistically rigorous analysis of a subset of comparable studies.

Once researchers have scoped their review appropriately for meta-analysis, they may choose to register with a peer review group, or at least follow the published guidelines of such a group. The choice of which guidelines to follow should be directed by careful consideration of both the substantive topic of the review, and the likely methodological issues that may arise in the review process. Such consideration is necessary because review groups may differ in their recommendations, both for specific methodological issues and for the general review process.

**Figure 2:**
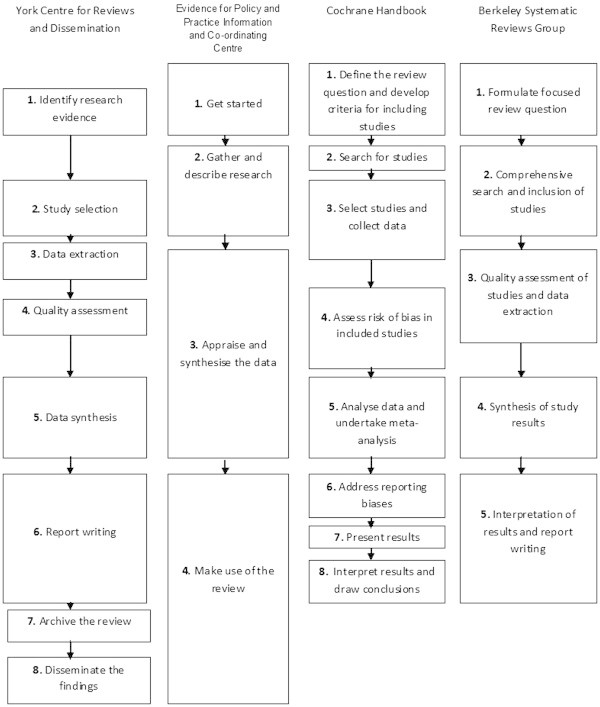
**Steps in a meta-analysis.** Note: These steps were taken from the contents section of the relevant handbook.

Figure 2 presents a summary of the steps defined by a number of distinguished review coordinating groups and experts. The Cochrane Collaboration is a premier medical systematic review co-ordinating group and peer review team that publishes a regularly updated Handbook for systematic review and meta-analysis procedures (Higgins & Green, 2011). The Cochrane Handbook is recommended by the Campbell Collaboration, a related review coordinating group focusing on social sciences. Other organisations also publish guidelines on conduct and methods for systematic reviews, including the York Centre for Reviews and Dissemination, the Evidence for Policy and Practice Information and Co-ordinating Centre (EPP-I Centre) at the University of London, and the Berkeley Systematic Reviews Group at the University of California. The figure collates information based on the contents sections of each organisation’s publication, with the assumption that the contents would provide some indication of what each organisation considered to be the primary sections of a review, and roughly in what order they should be considered.

As seen in Figure 2, even a small survey of these very well recognised groups produces a range of instructions regarding where to start, where to finish, the number of steps involved, what those steps consist of, and what is the best order for review stages. Each group gives a starting point, and agrees that synthesis is a key step. However, the recommendations differ in detail, especially on the archiving and dissemination activities expected of the reviewers. This difference in focus is partially due to the differing concerns of each discipline. Meta-analyses in medicine (the focus of the Cochrane Handbook) are aimed primarily at informing medical practitioners’ decision making (Higgins & Green, 2011), and as such focus on homogeneity of included sources and dissemination of treatment efficacy. In contrast, meta-analyses in social sciences and ecology may focus on identifying and describing heterogeneity, for the purposes of understanding the causal processes at work in the phenomenon under study (Koricheva et al. 2013). These differences in focus give rise to diverse perspectives on problems and, subsequently, can provide multiple “lenses” through which to view issues in meta-analysis.

### Issue 2. Appropriate eligibility criteria for primary studies

#### 2.1. Eligibility criteria for study designs

Systematic reviews in social research often encounter non-randomised evaluation designs. In social sciences, trials with a representative population may be considered more valuable than laboratory studies because of their ecological validity (Sampson, 2010). In addition, it is often not ethical or legal to randomise assignment to treatment and comparison conditions in social science experiments (Baunach, 1980). Furthermore, practical constraints may limit the implementation of such experiments, especially if they require the co-operation of multiple agencies which can be time- and resource-intensive to establish and maintain. Therefore, trials of new interventions in many areas of social science are often quasi-experimental or interrupted time series, or simple correlation designs.

As such systematic reviews in social science disciplines may need to deal with a variety of primary study designs. The medical literature generally advises against combining field trials with laboratory trials, or combining non-randomised designs with randomised designs (Higgins & Green, 2011; Shadish & Myers, 2004). One reason forwarded in support of the separation of randomised and non-randomised designs in meta-analysis is based on randomisation as a quality indicator; that is, evaluations using randomised designs must be of high quality, and therefore more likely than non-randomised (low quality) designs to show a real effect. However, treatment randomisation does not prevent difficulties of causal inference. Non-random sampling, differential attrition, and lack of treatment integrity can introduce alternate explanations for treatment effects even in randomised trials (Littell, 2004; Sampson, 2010; Shadish et al. 2002). Therefore, some authors argue, the selection of studies should be based on their likely validity in the context of the research question (Littell et al. 2008). Moreover, it is apparent that meta-analysis of a subset of available studies has the potential to lead to less accurate, biased or otherwise misleading results. These factors have led reviewers to follow a ‘best available’ inclusion strategy when selecting study designs to combine in a meta-analysis (Higgins & Green, 2011), or to use a combination of narrative review and meta-analysis to attempt to cover the range of evidence on a topic (Stewart et al. 2012). In general, this issue appears to be approached on an ad hoc basis, and methods for combining studies with different evaluative designs are still being developed.

The above discussion suggests that when conducting a systematic review on any question, all of the likely sources of bias in a corpus of studies should be considered before deciding whether or not to exclude studies based on randomisation. In order to obtain the best possible estimate of an intervention’s effectiveness, it may be necessary to review sources that investigate the problem using multiple methodologies. It is therefore useful to include conclusions from qualitative studies, information about treatment integrity and difficulties of implementation, and other non-empirical information arising from primary studies in a narrative review section that complements the meta-analysis results. Social researchers have developed detailed methods for conducting this type of mixed-methods systematic review (Harden & Thomas, 2005).

#### 2.2 Eligibility criteria for outcome measures

In any evaluation, different outcomes are considered to be important by different stakeholders. In criminology, for example, reoffending is a quantitative outcome valued by police, while politicians may be more interested in public satisfaction with a particular intervention process. Scientists may also seek information on outcomes relevant to their theoretical model of the intervention effect, and process evaluation and cost-benefit analysis require further relevant but very different outcomes. In addition, each of these outcomes may be measured in a number of ways. Selecting relevant outcomes, therefore, is one hurdle that needs to be addressed in any meta-analysis.

A second hurdle is how to deal with studies that report multiple relevant outcomes based on the same sample. Large-scale social experiments may capitalise on the rare data collection opportunity by collecting information on a very wide range of outcomes. The number of outcomes thus arising from a single trial presents a challenge for a reviewer seeking the most relevant measure of intervention effectiveness across multiple studies. This challenge may extend to a number of issues: multiple different measures of the same construct within a study, a single study providing distinct outcomes measured in different units, and a lack of consistency among studies concerning what outcomes should be measured and how to measure them.

As illustration, these considerations raised a number of questions in the policing review of Mazerolle et al. ( 2013). Their questions included the following. Can we combine reoffending with participant satisfaction in an evaluation of policing programs? Can we combine participants’ ratings of satisfaction and perceived fairness of the process? Can we combine two different studies’ measures of satisfaction, if they use different questions with different scales and if so, how is such a combination to be achieved? If we don’t combine all the outcomes, should we just pick one? How do we decide which is important and which to discard? What about the studies that do not report on the outcomes we selected but still evaluate the program; should their information just be lost? If we want to investigate multiple outcomes, is it legitimate to perform multiple meta-analyses, in light of concerns about multiple testing and correlated outcomes? Questions like these are not limited to criminology, but may arise more often in research fields where data collection is complex or difficult, such as international development (Waddington et al. 2012). A potential solution, as employed in a systematic review of violent crime interventions in developing countries (Higginson et al. 2013), is to use a series of meta-analyses to test specific outcomes within a broader narrative review of outcomes more broadly defined.

A related issue is non-standard measurement of outcomes. Primary studies may present differences in terminology and operational definitions, failure to provide scale validity and reliability scores, and heuristic measurement methods. For example, these problems were observed by Mazerolle et al. ( 2013) in a review of policing programs, where public perception of the police was a key outcome. One reviewed study (Skogan & Steiner, 2004) measured perceptions of police with ten items assessing dimensions of police demeanour, responsiveness, and performance, while another (Hall, 1987) measured perceptions of police using a single item: ‘The Santa Ana Police Department is effective’. A third study (Ren et al. 2005) identified confidence as a key outcome and measured it with seven items asking whether officers were fair, courteous, honest, not intimidating, worked with citizens, treated citizens equally, and showed concern; while a fourth (Murphy et al. 2008) used four items measuring confidence in police, police professionalism, whether police do their job well, and respect for police, and called that legitimacy. Some authors had reported statistics for individual items (e.g., Sherman et al. 1998) while other authors had only reported statistics for an aggregate scale (e.g., Ren et al. 2005).

In some fields, where key outcomes are latent and must be observed through constructed questionnaires, this difficulty is obvious. In fields where outcomes are observed more directly, their selection and combination may be more straightforward. Nevertheless, examples of this issue can be found across many disciplines: in biology, plumage colouration could be measured with melanin-based or carotenoid-based coloration, further split into saturation, brightness, and hue (Badyaev & Hill, 2000); in medicine, treatment effectiveness could be measured with survival or quality-adjusted life years (Grann et al. 2002) education interventions could be assessed with students’ social and emotional skills, attitudes, social behaviour, conduct problems, mental health, or academic performance (Durlak et al. 2011), for example. The common difficulty faced by reviewers across different disciplines is how to identify, select, and combine relevant outcomes for a meta-analysis.

Across the disciplines, different methods have been put forward for resolving the difficulty of identifying and combining relevant outcomes. One possibility is to simply combine outcomes into one large estimate of treatment effect. However, there is an inherent danger in this approach, of combining outcomes that may be affected in different directions by the treatment, producing an overall effect size that is somewhere in the middle but does not provide a full picture of the intervention’s effects. In addition, the interpretation of such an effect size may be very difficult. In ecology, some researchers argue that each question demands a unique outcome measurement that is defined by the research question and the context of the ecological process (Osenberg et al. 1999).

In social sciences, many reviews present multiple effect sizes for different outcomes separately (e.g., Hedges et al. 1994). This approach aids clarity of interpretation, but it also presents two key difficulties. The first is the selection of appropriate outcomes to present. In a field where studies may measure a great number of outcomes for a single intervention, it is untenable to present separate effect sizes for them all – the huge number of outcomes would erase any previous advantage in ease of interpretation. Second, and more seriously, testing a very large number of outcomes for significance of effect size raises the probability of a Type 1 error beyond the acceptable threshold. Finally, in most cases the multiple outcomes are unlikely to be independent of one another, in which case presenting multiple outcome effect sizes may mislead as to the true effectiveness of an intervention.

Sophisticated statistical methods have been put forward to deal with the problem of multiple outcomes. For example, multivariate approaches demonstrated by Becker ( 2000) provide estimated correlations among outcome measures as well as a correction for missing outcome measures in some trials. These methods address many of the concerns above. Unfortunately, they are rarely applied by practitioners who encounter these issues in a review. Reasons for this lack of application may include a lack of awareness about the availability of these methods, or their increased statistical complexity. Additionally, the information required by these approaches may not be available in primary research in areas where data reporting requirements are not standardised (see the following section of this paper). For example, multivariate meta-analysis requires estimates of the correlations among outcomes reported within each study, information that is very rarely available (Riley, 2009). As computational technology, statistical familiarity, and data reporting standards improve, these solutions hopefully will become more accessible, and thus more widely used. At present, the recommendation of the Campbell Collaboration is simply to use ‘some procedure’ to account for outcome dependence, and to provide detailed description and justification for the choice of procedure (Becker et al. 2004).

In light of the above discussion, the most sensible course for reviewers appears to be to decide a priori what outcomes are important and what definitions count towards each outcome, as is recommended by the Cochrane Collaboration (Higgins & Green, 2011). Reviewers can seek out the opinions of experts in the field of primary research to help determine what outcomes are useful. It is also suggested that reviewers consult a methodology expert to help determine which outcome measures are feasible to combine and how best to account for non-independence among outcomes. These consultations may be most helpful at the scoping stage of the review.

### Issue 3. Data reporting in primary studies

Reporting standards vary among journals, and more generally among unpublished theses and reports. Each evaluation can have a different style for reporting analysis strategy and results, study design, and implementation issues. Many studies focus solely on statistical significance of the results, while others only report descriptive statistics. In addition, studies may report results only as “not significant”, and omit test statistics, descriptive statistics, or direction of effect thereafter.

This problem has been remarked upon by reviewers in many disciplines (Johansen & Gotzsche, 1999; Lipsey et al. 2006; Littell, 2004). Reporting guidelines now exist for randomised controlled trials (CONSORT) (Schulz et al. 2010), observational studies (STROBE) (Von Elm et al. 2007), non-randomised evaluation studies (TREND) (Des Jarlais et al. 2004), economic studies (Mason & Drummond, 1995), psychological studies (American Psychological Association, 2010), self-report data (Stone & Shiffman, 2002), and animal studies (Kilkenny et al. 2010). Online databases, such as the EQUATOR website (Simera et al. 2010) and the MIBBI project (Taylor et al. 2008), have been established to provide regularly updated lists of reporting guidelines and minimum information checklists. These guidelines are useful resources for reviewers seeking to understand the type of information that might be reported in primary studies in their field. Whether this information is actually reported, however, is not guaranteed, and may vary by discipline. For example, most psychology journals require articles to be submitted according to American Psychological Association guidelines, including a minimum reporting standard for methods and results; in contrast, biology journals have a wide range of reporting requirements, which vary from journal to journal. In most disciplines, it is likely that reviewers will encounter the issue of incomplete data reporting at some point.

To address the issue of incomplete data reporting, it may be possible to contact authors for further clarification and data regarding their analyses. Where this fails it may be possible to make assumptions about the direction of effect or the experimental design based on the information provided in the document. In some cases it is feasible to back-transition from test statistics to obtain a measure of effect size, using procedures outlined in the meta-analysis texts of (Borenstein et al. 2009), Lipsey & Wilson ( 2001), and others. None of these procedures, however, can address the ambiguous direction of effect that may result from the primary study reporting a statistical test as “not significant”.

A key consideration, especially when dealing with effect sizes that are not reported because the test statistic was “not significant”, is the potential bias introduced by simply discarding studies with incomplete information. Studies are presumably more likely to fail to adequately report an effect size when it is close to zero, than when it is relatively large (or at least statistically “significant”). Discarding these studies, then, is likely to result in an upwardly biased overall meta-analysis estimate, because the included effect estimates will be, in aggregate, higher than the excluded ones. A similar problem may occur when authors attempt to treat incomplete reporting as missing data, and to estimate values for the missing data using a standard imputation procedure. Most standard imputation procedures assume that missing data are missing at random (Enders, 2010), and this assumption does not hold for missing studies due to incomplete reporting in meta-analysis. The probability of a study effect estimate being missing due to incomplete reporting is directly related to the value of the missing number, with missing numbers likely to be smaller than observed ones. Thus, any imputed data are likely to be upwardly biased, and thereby bias the overall meta-analysis result.

The issue of inconsistent data reporting can most satisfactorily be addressed by the areas of primary research. It is mentioned here as a commonly encountered and highly frustrating problem in meta-analysis in many fields. For this reason, systematic review teams are advised to engage a statistician to help with complex effect size calculations. Meta-analysis reports should record in detail which studies were excluded due to incomplete data, and exactly what calculations were used to compute effect sizes within each study, and including this information as an appendix to the meta-analysis report (e.g. as done in Mazerolle et al. 2013). Whatever alternative is taken up, the results may be validated by assessing the sensitivity of the overall meta-analysis to the method of dealing with missing and incomplete data.

### Issue 4. Sources of heterogeneity

One of the primary research questions in social science meta-analysis is how the effects of a particular treatment or intervention differ across key variables of interest; for example, are the effects of school-based drug prevention programs different for schools in low-income and high-income areas, or for pupils of different ages, or genders? Meta-analysis offers a unique opportunity to explore the answers to these questions by comparing treatment results across a range of studies. However there can be difficulties in determining what effects are due to the treatment or intervention studied, and what effects are due to study-level variables such as study design, measurement methods, or study sample. This has been identified as an issue in many disciplines including social sciences (Lipsey, 2003), epidemiology (Boily et al. 2009), and ecology (Osenberg et al. 1999).

A serious issue may arise when sources of heterogeneity in effect sizes are difficult to isolate. For example, if most studies using a particular variation on an intervention also use a particular measure of the intervention effect, it may be difficult to separate the effect of the intervention variation from potential artefacts of the measurement method. In a standard regression, predictor variables that vary systematically are referred to as “confounded”. This terminology is adopted for the purposes of the following discussion; specifically, study-level characteristics that vary systematically to some degree are considered “confounded” (Lipsey, 2003).

Detecting confounded moderators may be straightforward in a meta-analysis of few studies. A table of key characteristics may be sufficient to reveal the degree to which study characteristics vary systematically (e.g. Mazerolle et al. 2013). In a larger sample of studies, however, this type of confounding may be more difficult to detect, and may result in inaccurate results of meta-regression (Viechtbauer, 2007) and other heterogeneity tests. Visualisation tools, such as meta-regression plots, may be useful in attempting to detect confounding. Comparing the results of meta-regression analyses with a single predictor to meta-regression with multiple predictors may also help to reveal the degree of confounding present. If study-level variables are good predictors of effect size when entered in to meta-regression as a series of single predictors, but their predictive power diminishes when entered as multiple predictors in the same meta-regression, then the variables may be correlated and potentially confounded.

A range of options have been posited for dealing with confounding once it has been detected. Basic options include meta-regression, subgroup analysis, and random-effects models, which are discussed in most meta-analysis texts (e.g. Borenstein et al. 2009; Koricheva et al. 2013; Sutton et al. 2000). More complex and statistically demanding options include network meta-analysis (Lumley 2002), Bayesian meta-analysis (Sutton & Abrams, 2001), and individual level data meta-analysis (Riley et al. 2010). The choice of which approach to use will depend on the amount and quality of data available, the degree and nature of confounding, the aims and scope of the research question, and the capabilities of the review team. Ultimately, some datasets may be deemed inappropriate for meta-analysis, but experts recommend attempting to correct for heterogeneity before abandoning meta-analysis altogether (Ioannidis et al. 2008). In particular, for research areas where the number of studies is limited by time (e.g., longitudinal research) or resources (e.g., large scale social interventions), this issue is likely to arise and may require more attention from specialists in meta-analysis methods.

## Conclusions

This paper has summarised guidance from a wide range of sources on how to deal with four issues that may be encountered in systematic reviews and meta-analysis of applied social science research. A review of methods literature from statistics, ecology, psychology, epidemiology, and criminology, has compiled a set of resources for the consideration of researchers who may encounter these issues in the process of undertaking their own systematic review and meta-analysis.

One way that reviewers can address these issues broadly is to ensure that the review team or advisory group includes members from multiple disciplines. A helpful advisory group, perhaps in addition to the core systematic review team, may include at least one methods expert and at least one expert on each substantive area that may have a bearing on the review question. The advisory group can be consulted at each stage of the research, in particular, in scoping the review question, and in any methodological decision making.

Experienced reviewers encountering one or more of these issues may be tempted to dismiss the entire research question as unanswerable with current methods. Indeed, in many situations this reaction may be perfectly appropriate. However, other experts may argue that in some situations, imperfect synthesis is better than none at all, particularly when a review is requested for the purposes of policy guidance. This paper is intended as a resource to direct applied researchers to possible resolutions for practical issues that may be encountered when attempting to use meta-analysis to address unanswered questions in their field. It is also intended for researchers attempting meta-analysis for the first time, who may attempt to address these issues with ad hoc resolutions if they are unaware of where to look for other methodological guidance. We therefore call for more attention to these issues from methodology experts, and more communication between applied researchers who have previously addressed these issues within their own discipline.
